# Headspace analyses using multi-capillary column-ion mobility spectrometry allow rapid pathogen differentiation in hospital-acquired pneumonia relevant bacteria

**DOI:** 10.1186/s12866-021-02102-8

**Published:** 2021-02-28

**Authors:** Nils Kunze-Szikszay, Maximilian Euler, Martin Kuhns, Melanie Thieß, Uwe Groß, Michael Quintel, Thorsten Perl

**Affiliations:** 1 Department of Anesthesiology, University Medical Center Göttingen, Robert-Koch-Straße 40, 37075 Göttingen, Germany; 2 Institute for Medical Microbiology, University of Göttingen, Kreuzbergring 57, 37075 Göttingen, Germany; 3 Institute of Plant Science and Microbiology, Molecular Plant Genetics, University of Hamburg, Ohnhornstraße 18, 22609 Hamburg, Germany; 4 Department of General, Visceral and Pediatric Surgery, University Medical Center Göttingen, Robert-Koch-Straße 40, 37075 Göttingen, Germany

**Keywords:** Pneumonia, Microbiological techniques, Volatile organic compound, Metabolite, Ion mobility spectrometry

## Abstract

**Background:**

Hospital-acquired pneumonia (HAP) is a common problem in intensive care medicine and the patient outcome depends on the fast beginning of adequate antibiotic therapy. Until today pathogen identification is performed using conventional microbiological methods with turnaround times of at least 24 h for the first results. It was the aim of this study to investigate the potential of headspace analyses detecting bacterial species-specific patterns of volatile organic compounds (VOCs) for the rapid differentiation of HAP-relevant bacteria.

**Methods:**

Eleven HAP-relevant bacteria (*Acinetobacter baumanii, Acinetobacter pittii*, *Citrobacter freundii*, *Enterobacter cloacae*, *Escherichia coli*, *Klebsiella oxytoca*, *Klebsiella pneumoniae*, *Pseudomonas aeruginosa*, *Proteus mirabilis*, *Staphylococcus aureus*, *Serratia marcescens*) were each grown for 6 hours in Lysogeny Broth and the headspace over the grown cultures was investigated using multi-capillary column-ion mobility spectrometry (MCC-IMS) to detect differences in the VOC composition between the bacteria in the panel. Peak areas with changing signal intensities were statistically analysed, including significance testing using one-way ANOVA or Kruskal-Wallis test (*p* < 0.05).

**Results:**

30 VOC signals (23 in the positive ion mode and 7 in the negative ion mode of the MCC-IMS) showed statistically significant differences in at least one of the investigated bacteria. The VOC patterns of the bacteria within the HAP panel differed substantially and allowed species differentiation.

**Conclusions:**

MCC-IMS headspace analyses allow differentiation of bacteria within HAP-relevant panel after 6 h of incubation in a complex fluid growth medium. The method has the potential to be developed towards a feasible point-of-care diagnostic tool for pathogen differentiation on HAP.

**Supplementary Information:**

The online version contains supplementary material available at 10.1186/s12866-021-02102-8.

## Background

About one-quarter of all infections in intensive care medicine are episodes of hospital-acquired pneumonia (HAP) [1]. Behind central line-associated bloodstream infections, HAP is the second common infectious condition in the intensive care unit (ICU) [2]. Mechanical ventilation is the most important risk factor for HAP. Up to 27% of all patients ventilated > 48 h suffer from HAP. ICU and hospital stays are prolonged in these patients and mortality rates are markedly increased [3]. Eventually, HAP leads to sepsis and septic shock with mortality rates up to 50% [4]. Rapid and adequate antibiotic therapy contributes to improved outcomes in these patients [5, 6].

The judgement of adequacy of antibiotic therapy thereby still depends on the results of conventional microbiological diagnostics with mean turnaround times of 71 h from sampling to the final results [7]. During that time, both patients and intensive care practitioners are forced to rely on the adequacy of the initially chosen calculated broad-spectrum antibiotic therapy, which is based on the patient’s individual risk profile and the knowledge of local microbial characteristics and probability for antibiotic resistances [8]. Adaption of antibiotic therapy is based on the results of pathogen identification and antibiotic resistance testing. Thereby, both escalation and downgrading of antibiotic therapy should be carried out as early as possible to ensure effectiveness on the etiological pathogen and to avoid induction of antimicrobial drug-resistances.

Besides proteomic (e.g. MALDI-TOF-MS) and genomic (e.g. multiplex PCR) methods the detection of microbial volatile organic compounds (VOC) was recently introduced as a possible strategy for pathogen identification [9, 10]. Multi-capillary column-ion mobility spectrometry (MCC-IMS) is a highly sensitive analytical method enabling the detection of volatile substances down to a range of picograms per liter (parts per trillion). MCC-IMS allows the user to investigate complex and humid gas samples, such as headspace samples of bacterial cultures or even breathing air [11]. Recent studies did show the feasibility of MCC-IMS analyses to detect microbial VOC and to differentiate between species of human pathogenic bacteria and fungi [12–14].

Based on the results of our previous studies we assumed that differentiation of a panel of HAP-relevant bacteria might be possible already after 6 h of incubation. We conducted an experimental study to test this hypothesis on a panel of 11 HAP-relevant bacteria. Successful differentiation of such a panel after 6 h would confirm the application’s eligibility as an innovative diagnostic approach in the setting of intensive care medicine.

## Methods

### Growth medium and volatile background

All bacteria were grown in Lysogeny Broth fluid medium (LB, Carl Roth GmbH + Co. KG, Karlsruhe, Germany). A pH of 7.2 was adjusted at a temperature of 37 °C using standard Tris-HCl buffer. Aliquots of 100 ml of the medium were transferred into 250-ml culture flasks (Schott Duran, Mainz, Germany) and autoclaved for 25 min at a temperature of 121 °C.

To investigate the volatile background six measurements of the cleaned and autoclaved culture flasks and six measurements of the sterile LB medium were performed using both ion modes of the MCC-IMS. For this, sterile LB samples were agitated constantly at a temperature of 37 °C for at least 1 h before measurements.

Compounds that were detected in the measurements of sterile LB medium and/or the sole culture flasks were considered to be part of the volatile background. These compounds were neglected if they did not show any obviously visible changes. In case of doubt these VOCs were analysed. Newly occurring signals as well as signals with increasing or decreasing intensity were considered to be useful discriminators and were therefore analysed for the present study.

### HAP panel

For the HAP-specific pathogen panel, one culture of the following 11 bacterial strains was used: *Acinetobacter baumanii* (DSM 24110, German Collection of Microorganisms and Cell Cultures, DSMZ, Braunschweig, Germany), *Acinetobacter pittii* (DSM 103739), *Citrobacter freundii* (DSM 24120), *Enterobacter cloacae* (DSM 30054), *Escherichia coli* (DSM 1103), *Klebsiella oxytoca* (DSM 24121), *Klebsiella pneumoniae* (DSM 2026), *Pseudomonas aeruginosa* (DSM 1117), *Proteus mirabilis* (DSM 4479), *Staphylococcus aureus* (DSM 13661), *Serratia marcescens* (DSM 50904). All strains were stored at − 80 °C as glycerol stocks (LB *w/v* 55% glycerol).

For headspace analyses, overnight cultures were inoculated from glycerol stocks. From this culture, 100 μl were transferred into a 250 ml Schott flask containing 100 ml of LB fluid medium. Cultures were left constantly moving at a temperature of 37 °C for 6 h before headspace analyses were performed. Afterwards, each culture was grown in addition for 72 h on Columbia sheep agar plates and verified by MALDI-TOF-MS analyses using the Bruker BioTyper 3.0 system (Bruker Daltonics, Bremen, Germany). A single culture was grown for each headspace measurement. A flow chart of the experimental setup can be found as supplemental figure.

### Headspace sampling

Headspace analyses were performed after 6 h of incubation at 37 °C. For each species, six measurements were performed using the positive ion mode of the MCC-IMS and six measurements were performed using the negative ion mode.

For the headspace analyses, each culture flask was attached to the MCC-IMS using a two-way (in- and outlet) screw cap. A constant flow of 100 ml/min of synthetic air was applied via the gas inlet of the screw cap and the gas outlet was attached to the sample inlet of the MCC-IMS device. The MCC-IMS’s sampling loop was flushed with the gas sample for 30 s at a constant flow of 100 ml/min. After the sampling was completed, the culture flasks were disconnected, and incubation was continued until the next measurement could be started. In order to keep the sample loop clean, at least two humid air measurements were performed between the headspace analyses. The next measurement was performed after these measurements confirmed cleanliness of the system. Otherwise, more humid air measurements were performed.

### Multi-capillary column-ion mobility spectrometer

The specifications of the MCC-IMS device used for this study are summarised in Table 1. The principles of gas sampling analyses by MCC-IMS using a multi-capillary column (MCC) for pre-separation has been described in detail before [15]. The authors of this present study did also describe the working principle of this MCC-IMS device previously [13].

**Table 1:** Specifications of the MCC-IMS used for the study

MCC-IMS device	BioScout, B&S Analytik GmbH, Dortmund, Germany
Preseparation	Multi-capillary column OV-5 (length 22 cm), Multichrom Ltd., Novosibirsk, Russia
MCC temperature	40 °C
Sample loop	Stainless steel, volume 10 ml
Ionization source	ß-radiation, ^63^Ni (550 MBq)
Electric field strength	330 V/cm
Shutter opening time	300 μs
Drift and carrier gas	Synthetic air, Air Liquide AG, Düsseldorf, Germany
Drift gas flow	100 ml/min
Carrier gas flow	150 ml/min
Sampling time	30 s
Sample flow	100 ml/min
Temperature	ambient
Pressure	ambient
Tubing	PTFE, Bohlender GmbH, Grünsfeld, Germany

In the drift region of the IMS, ions move through an external electrical field that can be charged positively or negatively. In this study, headspace samples were investigated in both the positive and the negative ion mode.

### MCC-IMS data analyses

Analyses of the detected MCC-IMS signals were performed using the VisualNow software (Version 1.2, B&S Analytik GmbH, Dortmund, Germany). The software visualizes MCC-IMS data as a three-dimensional matrix in which the x-axis indicates the inverse ion mobility in volt seconds per square centimetre (Vs/cm^2^) and the y-axis indicates the MCC retention time in seconds (s). In the resulting topographic plot, the matrix values indicate the signal intensity (SI) in volts (V). In order to normalize all MCC-IMS data, alignment was performed according to Vautz et al. 2009 and Perl et al. 2010 [16, 17]. MCC-IMS signals were identified by comparing the detected signals with a database, including the reference data for 125 substances. For each substance, reference measurements were performed prior to this study as it has been described before [12]. The IUPAC names of the substances were used in this study. Signals we were unable to identify were named according to their position in the 2D-topographic plot as “P_x_y”, whereas “x” is representing the inverse ion mobility × 1000 and “y” is representing the MCC retention time (e.g. P_685_20 = 0.685 Vs/cm^2^; 20 s).

### Determination of the VOC patterns of the HAP panel

In order to identify all VOC signals that were potential discriminators between the bacteria of the HAP panel, all obviously visible changing peak regions were statistically analysed using Excel (Microsoft Excel Professional Plus 2010, Microsoft Co., Redmond, USA) and Prism 7 (GraphPad Software Inc., San Diego, USA). In cases of doubt if a peak region changed it was statistically analysed. Each signal was investigated for normal distribution using the Shapiro-Wilks test of normality. Significance testing was performed using an ordinary one-way ANOVA for normally distributed parameters and the Kruskal-Wallis test for non-normally distributed parameters. Thereby, the median signal intensity of each compound in the headspace above sterile LB growth medium was compared with the median signal intensity over each bacterial culture of the panel. *P*-values < 0.05 were considered to statistically significant.

## Results

**Fig. 1:**
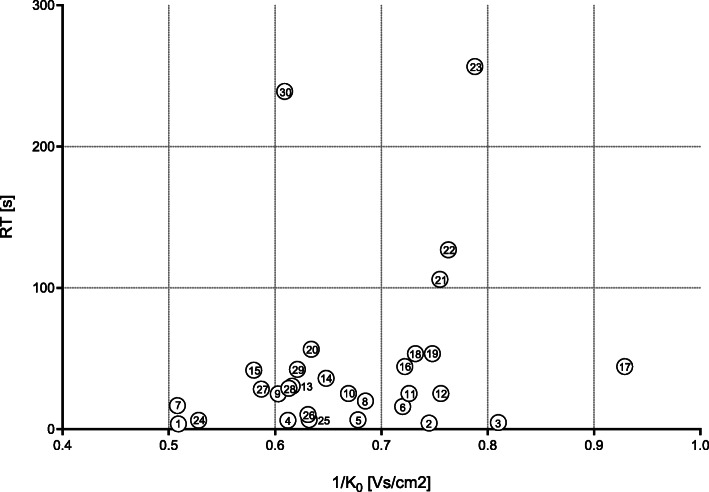
The positions of all VOC signals analysed in this study (positive and negative ion mode) in a 2D topographic plot with the x-axis indicating inverse ion mobility (1/K_0_ [Vs/cm^2^]) and the y-axis indicating the MCC retention time (RT [s])

**Fig. 2:**
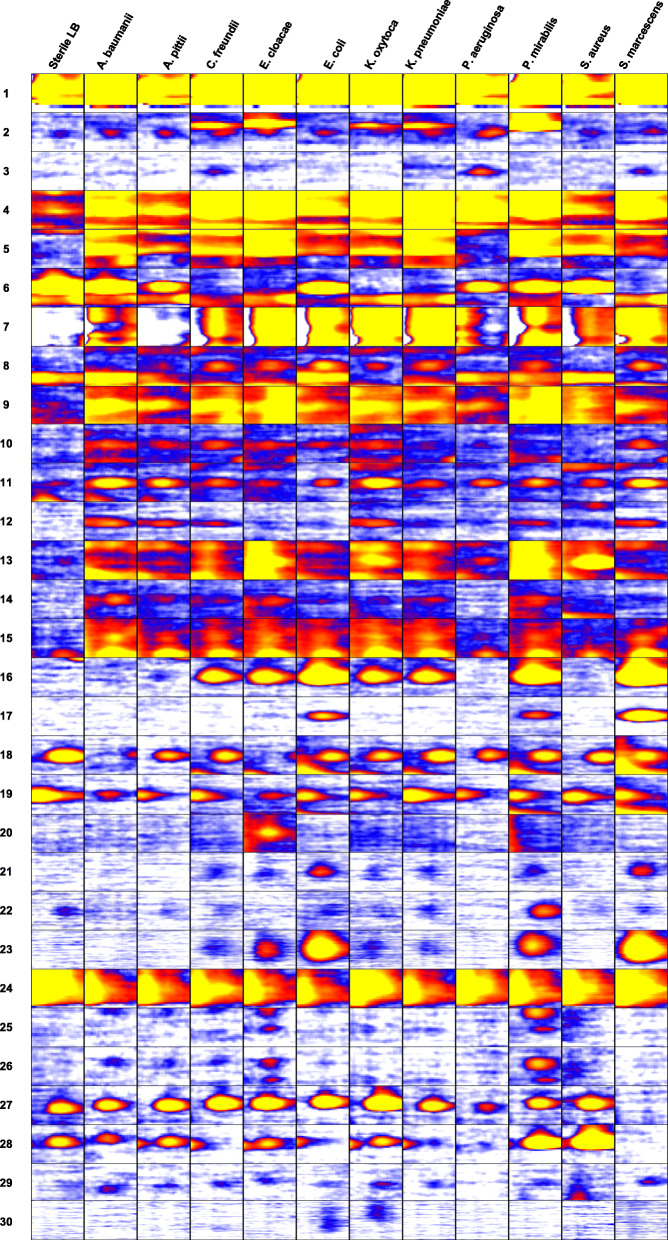
Images of all VOCs analysed in this study comparing the respective peak regions in all bacterial species of the HAP panel

After 6 h of incubation in LB, we detected changing VOC signals in the headspace of all bacterial species of the HAP panel. Significant changes in signal intensity in at least one of the bacterial species of the HAP panel were observed in 30 VOC signals (23 in the positive ion mode, 7 in the negative ion mode). Table 2 lists these VOC signals and Fig. 1 shows their positions in a 2D topographic MCC-IMS plot. Table 3 shows the median (min-max) signal intensities of all VOC signals for each species and summarizes the results of the statistical analyses. Figure 2 shows the peak regions of all VOC signals for all bacteria species.

**Table 2:** VOC signals that changed statistically significant in at least one bacterial species of the HAP panel

VOC No.	Substance name (or position)	1/K_**0**_ [Vs/cm^**2**^]	RT [s]
Positive ion mode			
1	Ethanol	0.509	3.7
2	P_745_4	0.745	4.3
3	P_810_5	0.81	4.7
4	P_612_6	0.612	6.3
5	P_678_7	0.678	6.7
6	P_720_16	0.72	16
7	P_508_17	0.508	16.7
8	P_685_20	0.685	19.9
9	P_603_25	0.603	25
10	P_669_25	0.669	25.2
11	P_726_25	0.726	25.2
12	P_756_25	0.756	25.2
13	2-Phenylacetaldehyd	0.616	30.4
14	P_648_36	0.648	36
15	P_580_42	0.58	41.8
16	Octan-1-ol (monomer)	0.722	44.3
17	Octan-1-ol (dimer)	0.929	44.3
18	Nonanal	0.732	53.5
19	P_748_54	0.748	53.5
20	P_634_57	0.634	56.6
21	P_755_106	0.755	106
22	P_763_127	0.763	127
23	Decan-1-ol	0.788	256.6
Negative ion mode			
24	P_528_6	0.528	6.3
25	P_632_7	0.632	6.8
26	P_631_10	0.631	10.3
27	P_587_28	0.587	28.4
28	P_613_29	0.613	29
29	P_621_42	0.621	42.3
30	Indole	0.609	239

Unknown substances were named according to their position in the 2D-topographic plot as “P_x_y”, whereas “x” is representing the inverse ion mobility × 1000 and “y” is representing the MCC retention time (e.g. P_685_20 = 0.685 Vs/cm^2^; 20 s).

*1/K*_*0*_ Drift time in Ion mobility spectrometry [Vs/cm^2^], *RT* Retention time [s]

**Table 3:**
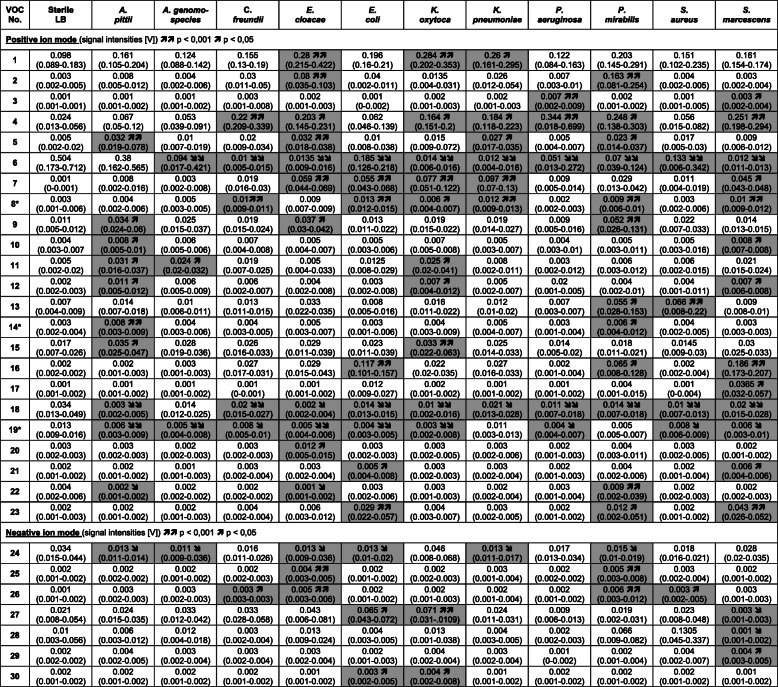
Median signal intensities of all VOC signals for each species

Signal intensities (SI) are given in volts. All values are median (min-max) values of the signal intensity (SI). Statistically significant changes in SI (compared to LB) are highlighted in gray with arrows indicating an increasing ( ) or decreasing ( ) trend in SI. Single arrows mark statistical significance with p-values < 0.05 and double arrows mark *p*-values < 0.001

**Fig. 3:**
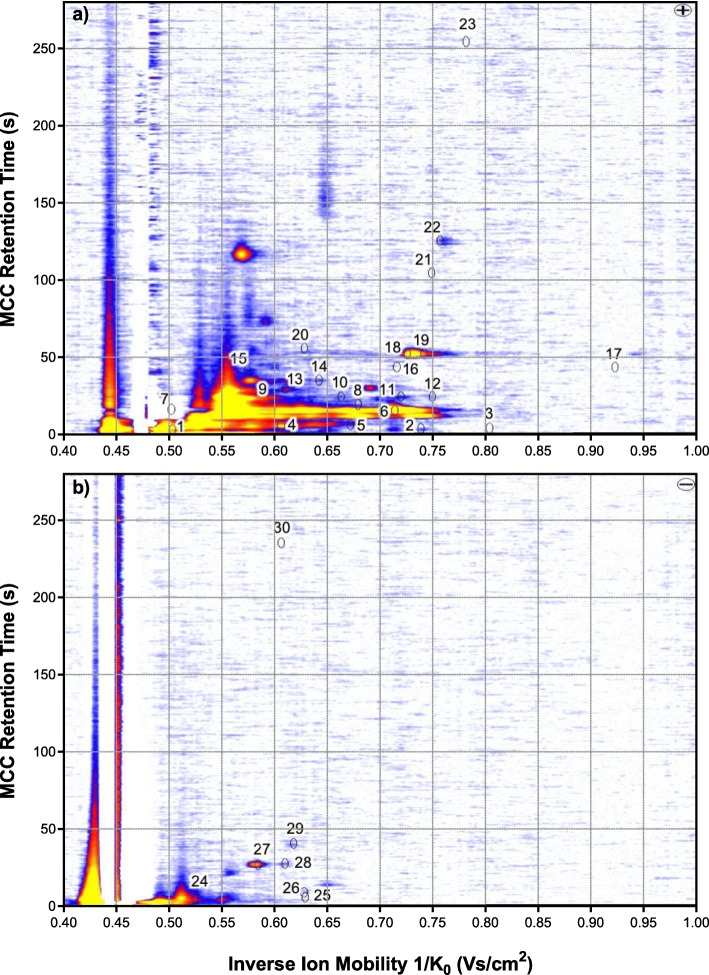
The positions of all 30 VOCs analysed in this study in a representative topographic plot of MCC-IMS measurements of the headspace over sterile LB medium in (**a**) the positive and (**b**) the negative ion mode of the MCC-IMS device. The remaining VOC signals and plot areas did not change during the experiments and were considered to be part of the volatile background

Signals that were detected in the headspace of sterile LB growth medium and that did not change obviously visible were considered to be part of the volatile background and therefore neglected. Figure 3 shows MCC-IMS plots of the headspace over sterile LB medium (positive and negative ion mode) with the positions of the VOC signals that were considered to differentiate the HAP panel. The remaining VOC signals and plot areas did not change during the experiments and were considered to be part of the volatile background.

With only four and five significantly changing VOC signals, *A. pittii* and *S. aureus* showed the least differences in the headspace analyses. In contrast, *S. marcescens* and *P. mirabilis* with 16 and 15 showed the highest numbers of significantly changing VOC signals. *E. cloacae, K. oxytoca, K. pneumoniae, P. mirabilis* and *S. marcescens* showed similar changes. Their VOC patterns were partly overlapping but still differed from each other in more than one VOC signal. They were therefore still considered differentiable among one another.

## Discussion

HAP is a significant cause of morbidity and mortality in intensive care medicine. Treatment outcome is positively affected by the fast onset of adequate antibiotic therapy [1, 6]. Besides genomic techniques (e.g. multiplex PCR) and proteomic applications (e.g. MALDI-TOF-MS), the metabolomic approach by using VOCs of microbial origin has been proposed for bacterial species differentiation [9]. MCC-IMS technology using multi-capillary columns for pre-separation of complex and humid gas samples has been used in several studies focusing on the detection of VOCs of microbial origin. Its ability for differentiating several human pathogenic microbes was described in both in vitro and in vivo experiments [12, 14, 18].

Although pre-analytical culturing and incubation is mandatory in respiratory samples, MALDI-TOF-MS is often used in medical microbiological laboratories resulting in a significant shortening of result turnaround times [19]. However, the assessment of microbial samples in MALDI-TOF-MS is complex and specially trained personnel is needed. Due to investment costs, its dimensions and the complexity of its handling MALDI-TOF-MS is not suitable for point-of-care use in an intensive care unit or even in small or remote hospitals.

In contrast, MCC-IMS technology is rather inexpensive, robust and has the potential for significant miniaturization. Given a suitable application and intuitive user interface it could serve as a point-of-care diagnostic tool operated by the local staff of e.g. an intensive care unit. At the current state, MCC-IMS does not provide information on antimicrobial resistance. However, fast and reliable pathogen identification could result in a significant advantage in guiding the initial antibiotic therapy.

The results of this study show that MCC-IMS can differentiate between 11 bacterial species in a HAP-relevant panel of bacteria. Therefore, MCC-IMS technology has the potential to be developed towards becoming a valuable diagnostic tool. Several questions need to be investigated in this way. For instance, in this present study we neither investigated the changes in VOC composition with more than one pathogen present nor did we validate our results on real-life clinical samples. However, together with other recently published studies, our results underline the potential of the method as an innovative diagnostic microbiological tool [20].

Multiplex PCR devices are commercially available for point-of-care microbial diagnostics. In the context of HAP these systems can lead to significantly reduced result turnaround times. However, technical performance and sensitivity need to improve to justify the routine use of these expensive “sample-in, answer-out” cartridge systems [7, 21].

The panel used in our study represents the most relevant part of etiological pathogens in bacterial HAP [22]. Previous studies confirmed the ability of MCC-IMS headspace analyses to differentiate a large bacterial panel after 24 h of growth on agar plates [12]. Further investigations focused on the metabolites emitted during the incubation of fast-growing *E. coli* and the slow-growing *P. aeruginosa* in the complex fluid medium LB [13]. Based on these previous results we hypothesized that 6 h incubation time might be suitable for both slow- and fast-growing bacterial species. This assumption was confirmed by the results of this present study. Further investigations might focus on the earliest possible time point for their differentiation.

Assuming that the composition of bacterial VOC patterns depends on the nutrient medium used for growing bacteria it is not surprising that the results of this present study partly differ from findings of previous studies [23]. Jünger et al. 2012 used Columbia sheep blood agar plates for their study and incubation lasted 24 h before the headspace over the bacteria cultures was analysed. Only a few substances that occurred in their study were reproduced with LB as growth medium. Ethanol, indole and P_631_10 were found in both studies and showed a consistent association with the VOC patterns of the bacteria in the panel. Ethanol can be found in different amounts in the headspace of almost every bacterial culture. It therefore does not seem to be particularly helpful for pathogen differentiation. Quantification of the actual ethanol amount and statistical significance testing may however add relevant information to the question. Indole correlated in both studies with the growth of *E. coli* and *K. oxytoca*, a VOC commonly reported for both bacteria [24]. Until now it remains unknown which substance stands behind P_631_10. However, the substance occurred in both studies over growing cultures of *E. cloacae* and *P. mirabilis*. In the present study, it additionally occurred in the headspace of *S. aureus*. Although only three VOCs were reproducible, we still consider the results to be congruent. Despite substantial differences in the composition of the nutrient media and the methodological approaches in both studies, the results confirm the potential of MCC-IMS headspace analyses for the differentiation of clinically relevant bacteria.

Our present study did not show any inconsistent results compared to previous studies using MCC-IMS for microbial differentiation [12–14]. It confirms that even in a panel of 11 HAP relevant bacteria differentiation can be achieved after only 6 h of growth in a complex nutrient medium.

There are some methodological limitations to our present study. Although a major part of HAP-relevant bacteria was investigated, there are obviously more than these 11 species that can cause HAP. Future investigations should therefore include further pathogens causative for HAP. These investigations should lay a special focus on bacteria that rely on special growth requirements under in-vitro conditions (e. g. *H. influenzae* and *S. pneumoniae*). Furthermore, we did not investigate the headspace over cultures with more than one bacterial species. Tracheal and bronchial colonization is common in intensive care medicine and is one mechanism leading to presence of more than one pathogen in the e. g. a specimen of tracheal aspirate. Future studies should address this problem by investigating the most common combinations of bacteria and fungi in airway specimens.

The results of this study, and potential future studies addressing the problems mentioned above, should be tested in a clinical study investigating the feasibility of headspace analyses for early pathogen differentiation in HAP.

## Conclusions

MCC-IMS headspace analyses enabled differentiation of the 11 HAP relevant bacteria after not more than 6 h of growth in LB fluid medium. The limitations of our study need to be addressed in future investigations, but in principle, the method has the potential to be developed towards a feasible point-of-care diagnostic tool in intensive care medicine.

## Supplementary Information

**Additional file 1.**

## Data Availability

All original data are available at the corresponding author.
