# Supplementary material for: Headspace analyses using multi-capillary column-ion mobility spectrometry allow rapid pathogen differentiation in hospital-acquired pneumonia relevant bacteria
Source: BMC Microbiol. 2021 Feb 28;21:69. doi: 10.1186/s12866-021-02102-8 (PMC7916313; doi:10.1186/s12866-021-02102-8)
Supplement: Supplementary file 1 — Additional file 1. [file 12866_2021_2102_MOESM1_ESM.pdf]

Headspace analyses using multi-capillary column-ion mobility spectrometry allow rapid pathogen differentiation in hospital-acquired pneumonia relevant bacteria

<u>Supplementary Material</u>

Nils Kunze-Szikszay[1]*; E-Mail; nils.kunze@med.uni-goettingen.de

Telephone: 0049 551 3967707; Telefax: 0049 551 3966039

Maximilian Euler[1]; E-Mail: maximilian.euler@med.uni-goettingen.de

Martin Kuhns[2];E-Mail: mkuhns@gmx.de

Melanie Thieß[3]; E-Mail: melanie.thiess@uni-hamburg.de

Uwe Groß[2]; E-Mail: ugross@gwdg.de

Michael Quintel[1]; E-Mail: mquintel@med.uni-goettingen.de

Thorsten Perl[4]; E-Mail: tperl@gwdg.de

*corresponding author

[1] Department of Anesthesiology, University Medical Center Göttingen, Robert-Koch-Straße 40, 37075 Göttingen, Germany

[2] Institute for Medical Microbiology, University of Göttingen, Kreuzbergring 57, 37075 Göttingen, Germany

[3]Institute of Plant Science and Microbiology, Molecular Plant Genetics, University of Hamburg, Ohnhornstraße 18, 22609 Hamburg, Germany

[4] Department of General, Visceral and Pediatric Surgery, University Medical Center Göttingen, Robert-Koch-Straße 40, 37075 Göttingen, Germany

Caption supplementary figure: Flowchart illustrating the workflow of the experiments.

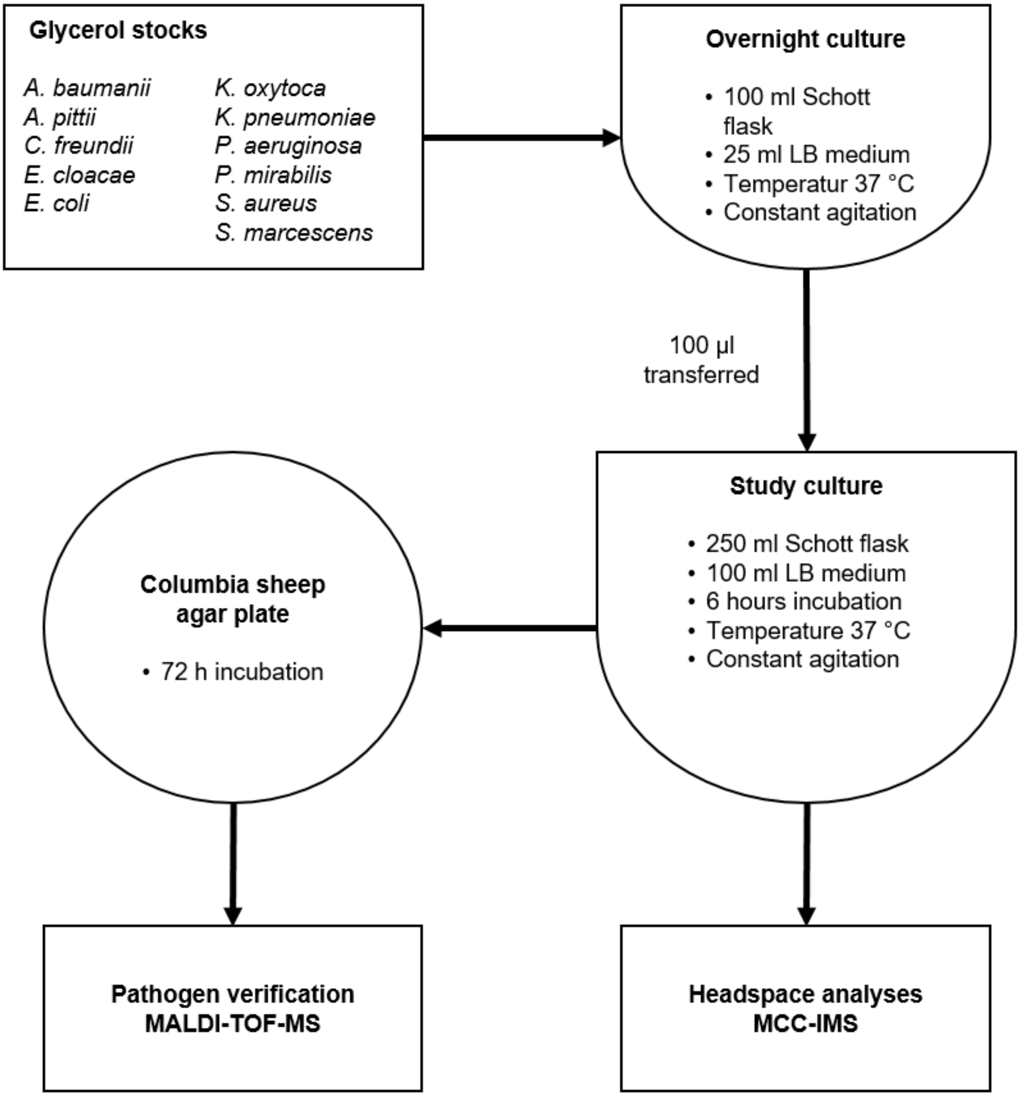
Glycerol stocks
A. baumanii
A. pittii
C. freundii
E. cloacae
E. coli
K. oxytoca
K. pneumoniae
P. aeruginosa
P. mirabilis
S. aureus
S. marcescens
Overnight culture
• 100 ml Schott flask
• 25 ml LB medium
• Temperatur 37 °C
• Constant agitation
100 µl transferred
Study culture
• 250 ml Schott flask
• 100 ml LB medium
• 6 hours incubation
• Temperature 37 °C
• Constant agitation
Columbia sheep agar plate
• 72 h incubation
Pathogen verification
MALDI-TOF-MS
Headspace analyses
MCC-IMS
